# Integrated transcriptomic and secretomic approaches reveal critical pathogenicity factors in *Pseudofabraea citricarpa* inciting citrus target spot

**DOI:** 10.1111/1751-7915.13440

**Published:** 2019-06-04

**Authors:** Yuheng Yang, Anfei Fang, Yang Yu, Chaowei Bi, Changyong Zhou

**Affiliations:** ^1^ College of Plant Protection Southwest University Chongqing 400715 China; ^2^ Citrus Research Institute Southwest University Chongqing 400712 China

## Abstract

Target spot is a newly emerging citrus disease caused by *Pseudofabraea citricarpa*. Outbreaks of this disease result in massive economic losses to citrus production. Here, an integrated study involving comparative transcriptomic and secretomic analyses was conducted to determine the critical pathogenicity factors of *P. citricarpa* involved in the induction of citrus target spot. A total of 701 transcripts and their cognate proteins were quantified and integrated. Among these transcripts and proteins, 99 exhibited the same expression patterns. Our quantitative integrated multi‐omic data highlight several potentially pivotal pathogenicity factors, including 16 unigenes that were annotated as plant cell‐wall‐degrading enzymes, 13 unigenes homologous to virulence factors from various fungi, and one unigene described as a small cysteine‐rich secreted protein, were screened and analysed. The screening of differentially expressed genes that encode secondary metabolism core enzymes implicated terpene metabolism in the pathogenicity of *P. citricarpa*. Overall, results indicated that plant cell wall degradation, plant–pathogen protein/polyribonucleotide interaction, and terpene biosynthesis have critical roles in the pathogenicity of *P. citricarpa*. This work demonstrated that integrated omic approaches enable the identification of pathogenicity/virulence factors and provide insights into the mechanisms underlying the pathogenicity of fungi. These insights would aid the development of effective disease management strategies.

## Introduction

**Figure 1:**
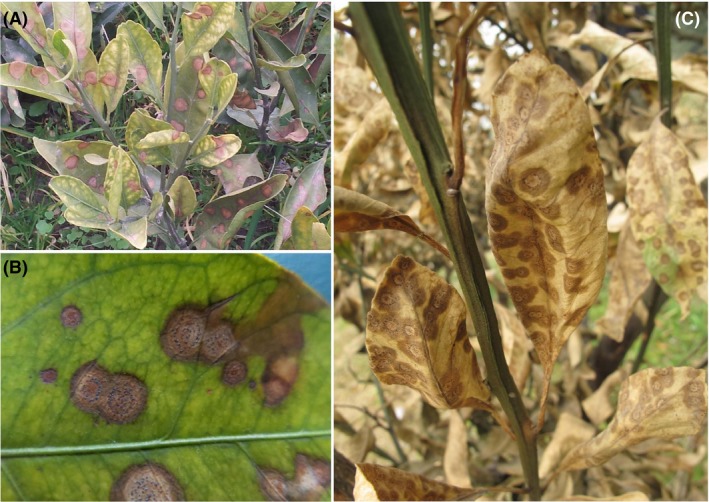
Symptoms of citrus target spot caused by *Pseudofabraea citricarpa*. A. View of infected shoots of Satsuma mandarin. B. Target spots on the leaf of Satsuma mandarin. C. Death of the whole tree caused by *P. citricarpa*.

Target spot, a newly emerging leaf‐spotting disease of Satsuma mandarin (*Citrus unshiu*) and kumquat (*Fortunella margarita*), has caused considerable economic losses in citrus production since it was first identified (Fig. 1) (Zhu *et al*., 2012). The causative agent of this disease was identified as *Cryptosporiopsis citricarpa* through Koch's procedure and morphological and molecular‐based phylogenetic characterization (Zhu *et al*., 2012). Subsequently, *C. citricarpa* had been redefined as the monotypic genus *Pseudofabraea* on the basis of its ITS rDNA sequence data (Chen *et al*., 2016). In contrast to other diseases that commonly affect young citrus leaves during warm and humid seasons, target spot occurs during late winter and early spring and causes severe leaf spotting or even defoliation (Yang *et al*., 2018). Therefore, understanding the complexity of the mechanisms responsible for the development of citrus target spot is crucial for improving the control and prevention practices of this infection.

secretomics are a global group of proteins that are constitutively secreted into extracellular spaces by cells, tissues, organs or organisms through known and unknown secretory mechanisms involving constitutive and regulated secretory organelles (Agrawal *et al*., 2010). The fungal pathogens of plants possess an extensive array of secretory proteins that are on the front line of host–fungus interactions (Lowe and Howlett, 2012). Fungal pathogens adapt to the host environment through the secretion of proteins and other molecules to facilitate nutrient acquisition and overcome immune responses (McCotter *et al*., 2016). These fungal species often deliver a set of effector proteins to facilitate host tissue colonization (Giraldo and Valent, 2013; Vleeshouwers and Oliver, 2014; Oliveira‐Garcia and Valent, 2015). Thus, deciphering fungal secretomics has become an important goal ever since secreted proteins were identified as the main effectors responsible for interactions between plants and fungi (Girard *et al*., 2013).

The signature and almost unique characteristic of microbial technology is the exceptional diversity of applications it can address (Timmis *et al*., 2017). Fungal secretomics have been long identified and analysed through polyacrylamide gel electrophoresis and bioinformatics prediction methods, which are constrained by their requirements for proteomic experimental approaches and published fungal genomic data (Bouws *et al*., 2008). The secretomics of fungal pathogens that lack published genomic information, however, are poorly known. The gradual application of integrated multi‐omic analysis to understand microbial biology has been enabled by the dramatic advancement of high‐throughput sequencing technology and the continuous improvement of bioinformatics methods (Fondi and Lio, 2015; Beltran *et al*., 2017; Alessi *et al*., 2018). The high‐throughput quantitative monitoring of the abundance of various biological molecules can provide complementary information for the discovery of valuable variations between components of different levels of the central dogma (Zhang *et al*., 2010; Wang *et al*., 2011). Here, we demonstrated how a combination of omic approaches helped us identify the pathogenicity factors and functional proteins of the necrotrophic fungi *P. citricarpa*.

In this study, we combined transcriptomic (RNA‐seq) and secretomic (iTRAQ) analyses to describe the differences between the gene transcript levels and protein levels of *P. citricarpa* under induction and non‐induction conditions at low temperatures. Our results showed that numerous genes/proteins are involved in plant cell wall degradation, protein interaction and secondary metabolite biosynthesis under induction conditions. Our findings provide further insight into the molecular mechanisms underlying pathogenic infections and can aid the development of highly effective control approaches for citrus target spot.

## Results

### Growth curve of *P. citricarpa*

Because the infection process of citrus target spot lasts for 9 months (Zhu *et al*., 2012), it is difficult to collect samples on infected citrus leaves directly. Thus, Satsuma mandarin leaf powder was adding to culture medium for inducing the expression of pathogenicity genes of *P. citricarpa*. During the first to seventh day of cultivation, the growth rate of mycelia decelerated, and mycelial production decreased. After the seventh day of cultivation, mycelial growth entered the logarithmic phase, and mycelial production gradually increased. The dry weight of mycelia reached the maximum value during the 16th and 19th days of cultivation but gradually declined after the 19th day of cultivation (Fig. S1). Therefore, for this study we selected the 17th day of cultivation as the sampling time point.

### Changes in the secretomic of *P. citricarpa* under induction treatment

**Figure 2:**
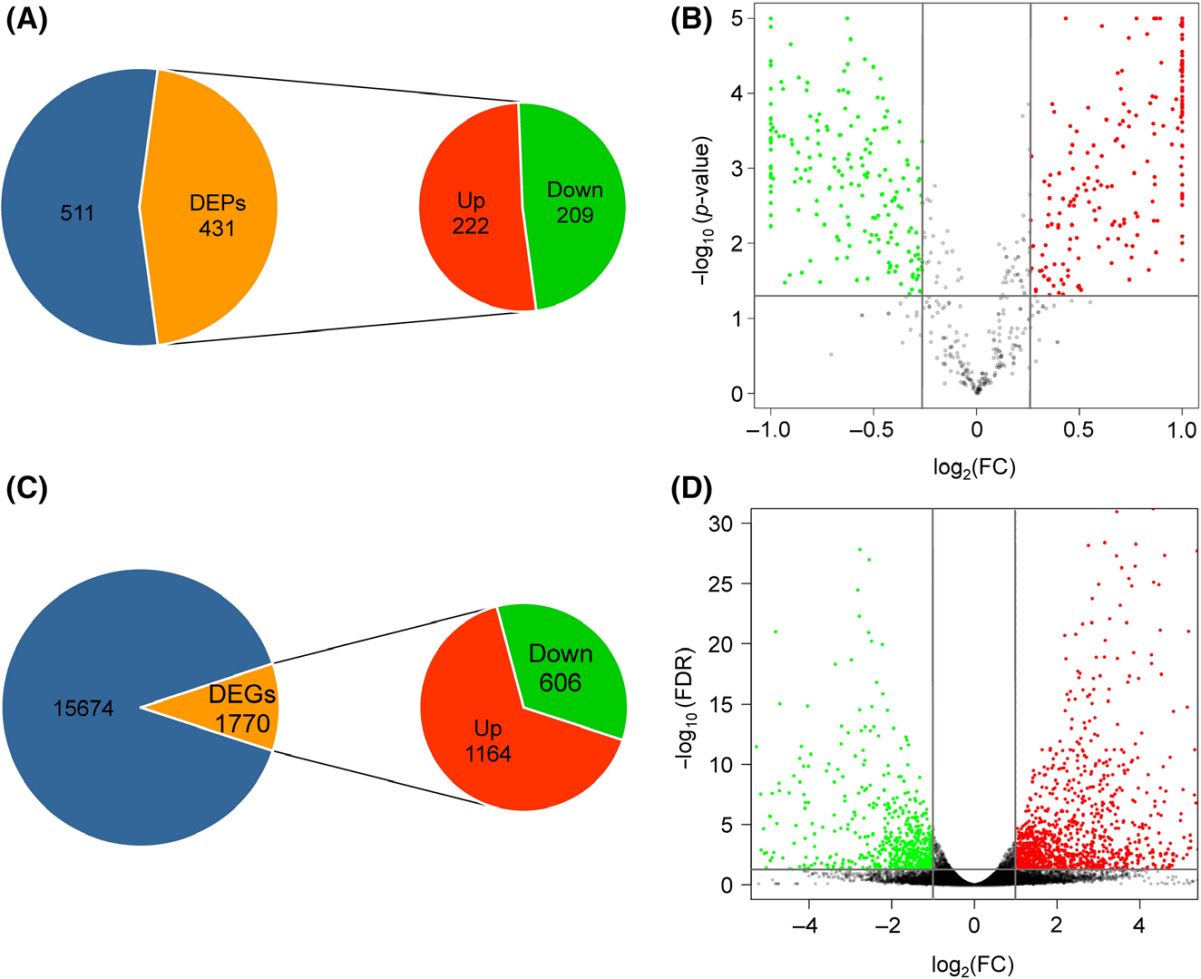
Annotation of the secretomic and transcriptomic data of *P. citricarpa*. A. Number of secretory proteins identified and quantified through the iTRAQ method. B. Differentially expressed proteins (DEPs) under induction treatment. C. Number of transcripts identified and quantified through the RNA‐seq method. D. Differentially expressed genes (DEGs) under induction treatment. The blue portions represent 511 secreted proteins (A) and 15 674 transcripts (C) in which expression is unchanged in inducing conditions relative to uninduced cultures.

Pathogenic fungi can secrete numerous proteins that are deployed to the host–pathogen interface during infection. We enriched and purified secretory proteins from *P. citricarpa* cultures grown in PDB medium with or without the Satsuma mandarin leaf powder inducer to illustrate the specific secretomic components that are expressed under induction conditions (Fig. S2). After labelling and nano‐HPLC‐MS/MS analysis, we identified 942 proteins, among which 701 were subjected to quantitative analysis (Table S1). Quantitative analysis provided a combined total of 431 proteins with drastically altered expression patterns under induction treatment relative to those under the control treatment as indicated by a fold change > 1.2 (*P *<* *0.05). These proteins included 222 upregulated and 209 downregulated DEPs (Fig. 2A).

Subsequently, we performed Gene Ontology (GO) analysis. Comparing the secretomic responses of *P. citricarpa* under induction and control treatments revealed that the annotated DEPs could be allocated to various functional categories (Fig. S3). Biological process annotation allocated 40.1%, 21.3% and 20.9% of the DEPs to metabolic process, single‐organism process and cellular process respectively. Cell component annotation identified 17.4%, 11.6% and 20.9% of the DEPs as proteins involved in cell component construction, organelles and cellular process respectively. Some DEPs were also enriched in membrane processes. Molecular functional annotation classified 45.2% and 22.0% of DEPs to catalytic and binding‐related activities respectively. Some DEPs that were involved in antioxidant activity were enriched.

The Kyoto Encyclopedia of Genes and Genomes (KEGG) pathway analyses categorized differentially accumulated proteins into 73 pathways, 12 of which were highly enriched. These highly enriched pathways included the alanine, aspartate and glutamate metabolism pathway; pentose phosphate pathway; carbon metabolism pathway; and peroxisome pathway (Fig. S4). Alanine, aspartic acid and glutamic acid metabolic pathways were the most drastically enriched pathways, and the highest number of unigenes was allocated to the carbon metabolic pathway (41 unigenes accounting for 14.39%). Thus, DEPs between the treatment and control groups were mainly involved in amino acid metabolism, energy metabolism and oxidation.

### Changes in the global transcriptomic of *P. citricarpa* under induction treatment

We cultured *P. citricarpa* separately in PDB media with or without an inducer to maximize the induced expression of proteins involved in pathogenic invasion. After removing citrus leaf genes, 526 516 raw reads were generated through transcriptomic sequencing and 17 444 expressed genes were assembled. The quantitative results yielded a combined total of 1770 DEGs with highly altered expression patterns with fold change ≥ 2 and FDR < 0.05 under the induction treatment relative to those under the control treatment. A total of 1164 upregulated DEGs and 606 downregulated DEGs were identified between treatments (Fig. 2C and Table S2).

We also performed GO analysis to compare the transcriptional response of *P. citricarpa* under induction and control treatments. Biological process annotation classified 13.2%, 11.7% and 11.0% of DEGs as proteins involved in metabolic process, single‐organism process and cellular process respectively. Cell component annotation classified 17.4% of DEGs as proteins constructed for cell part and cell, and some DEGs as proteins involved in macromolecular complex, organelle and membrane processes. Molecular function annotation categorized 14.3% and 8.2% of DEPs as proteins that participate in catalytic and binding‐related activities respectively, and some DEPs to transporter activity (Fig. S5).

The enrichment analysis of KEGG pathways was also conducted and indicated that DEGs mainly concentrated in tyrosine metabolism; valine, leucine and isoleucine degradation; nitrogen metabolism; fatty acid degradation; and peroxisome pathway (Fig. S6). The tyrosine metabolic pathway was the most drastically enriched pathway. The DEGs in two groups were mainly reflected in amino acid metabolism, energy metabolism and oxidation. These results conform to the results of secretomic analysis.

### Integrated analysis of secretomic and transcriptomic data

**Figure 3:**
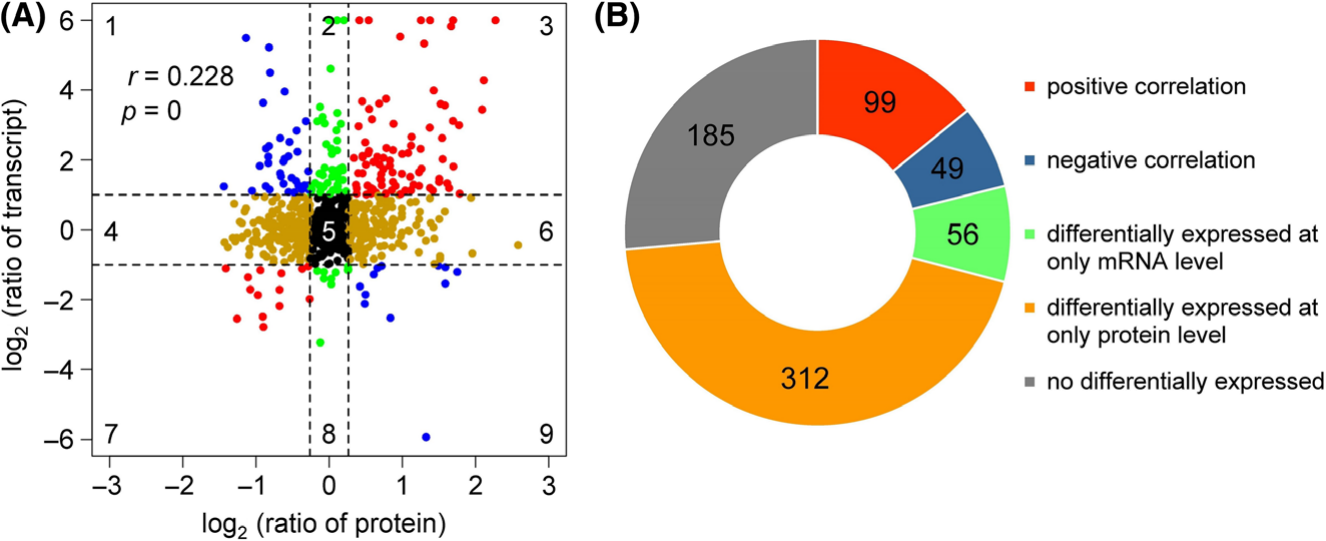
Correlation between the protein and transcript levels of the genes of *P. citricarpa*. A. Genes in quadrants 3 and 7 exhibited the same expression patterns on the transcript and protein levels, whereas those in quadrants 1 and 9 had different expression patterns on the transcript and protein levels. Genes in the other quadrants showed no difference in expression patterns on at least one level. *r*, Pearson's correlation coefficient; *P*,* P*‐value. B. Number of genes with positive (red), negative (blue) or no correlation on the transcript and protein levels.

We quantified changes in expression patterns on the transcriptional and translational levels to investigate the expression patterns of secretory proteins and transcripts related to critical pathogenicity factors. We quantified and integrated 701 transcripts and their cognate proteins (Table S3). We calculated Pearson's correlation coefficient (PCC) for transcriptomic and proteome data to further investigate their correlations (Fig. 3A). Several different types of gene expression patterns were found at the mRNA and protein levels. We found that 56 genes were drastically differentially expressed at the mRNA level but not at the protein level (Quadrant 2 and Quadrant 8) and that 312 genes were considerably differentially expressed at the protein level but not at the mRNA level (Quadrant 4 and Quadrant 6). In addition, 185 genes were not differentially expressed on the mRNA and protein levels (Quadrant 5), and the mRNA and protein expression levels of 148 of transcripts and their cognate proteins were drastically different (Quadrant 1 and Quadrant 9) (Fig. 3B).

**Figure 4:**
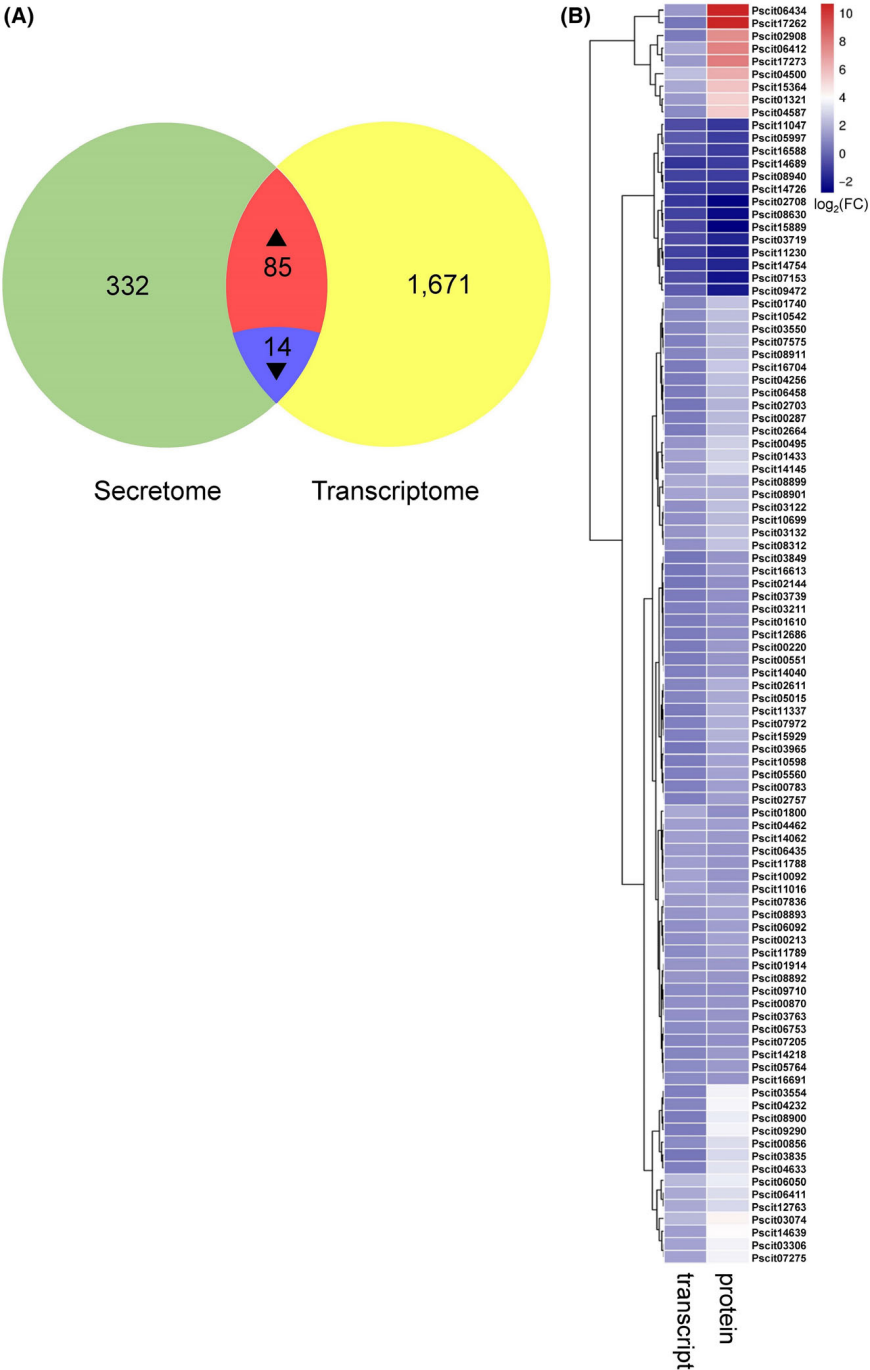
Expression dynamics and comparative analyses of differentially expressed proteins (DEPs) and differentially expressed genes (DEGs) in *Pseudofabraea citricarpa*. A. Venn diagram showing the regulated genes between the protein level and transcript level; (B) heatmap of genes with consistent expression trends in the secretomic and transcriptomic data of *P. citricarpa*. The *P*‐values of the 99 DEPs that satisfied fold change ≥ 1.2 between each sample were adjusted to < 0.05. The false discovery rates (FDRs) of the 99 DEGs satisfying fold change ≥ 2 between each sample were < 0.05.

Notably, 99 DEPs and DEGs had consistent expression trends, among which 85 were upregulated (Quadrant 7), and 14 were downregulated (Quadrant 7) (Fig. 4A). KEGG enrichment analysis indicated that most of the upregulated DEPs and DEGs were involved in glycosidic hydrolysis and energy metabolism, whereas downregulated DEPs and DEGs were mainly involved in lipid metabolism, secondary metabolite biosynthesis, glycolysis and aromatic compound degradation (Fig. 4B).

### Potential pathogenic factors of *P. citricarpa*

#### Cell‐wall‐degrading enzymes

**Figure 5:**
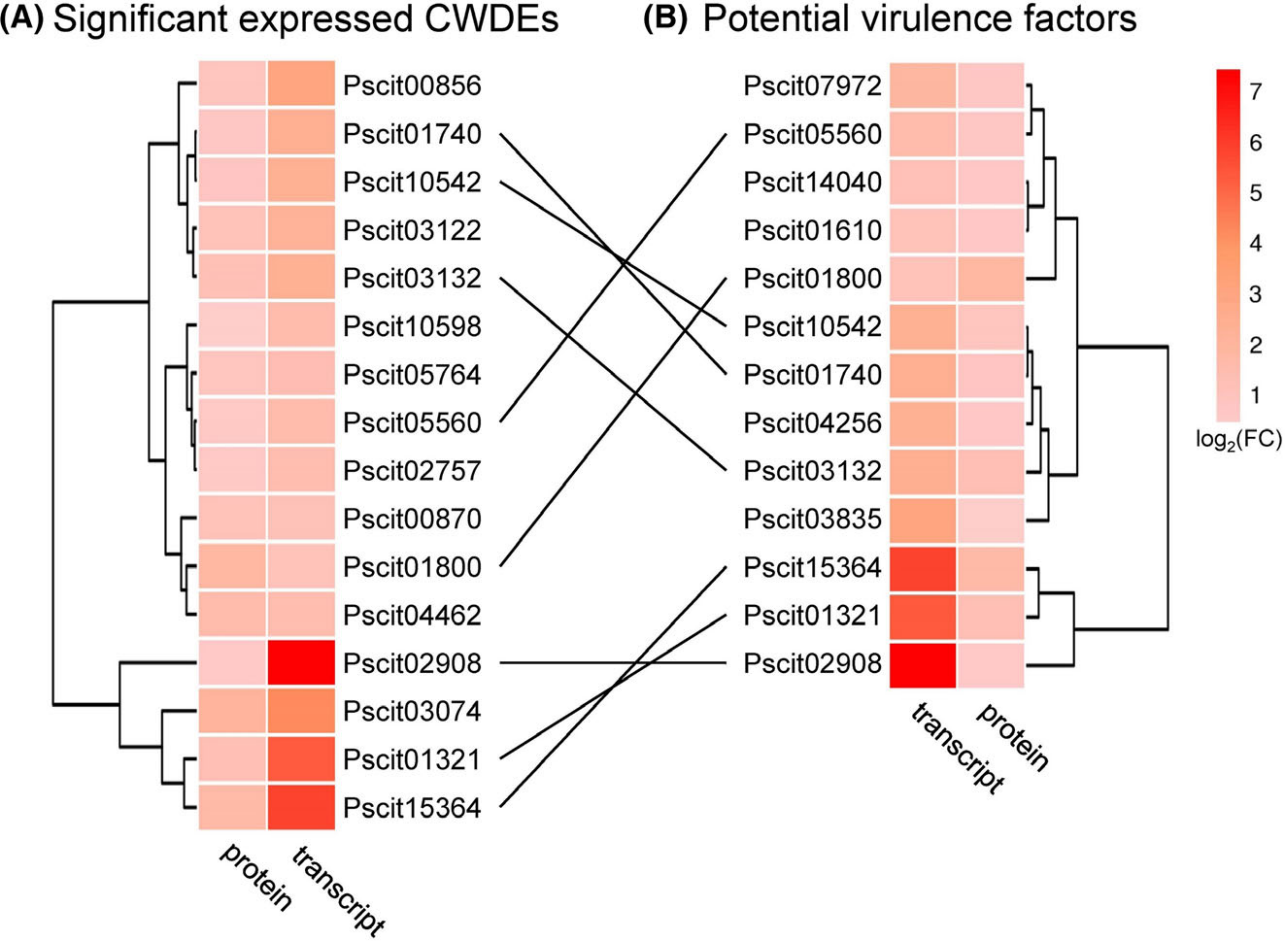
Heatmap of the DEPs and DEGs of P. citricarpa annotated as (A) plant cell‐wall‐degrading enzymes (CWDEs) and (B) potential virulence factors in plant–pathogen interaction. Eight of the DEGs and DEPs were annotated from the CAZy database and PHI‐base. The *P*‐values of the DEPs satisfying fold change ≥ 1.2 between each sample were adjusted to < 0.05. The FDR values of the DEGs satisfying fold change ≥ 2 between each sample were < 0.05.

Most plant pathogenic fungi produce cell‐wall‐degrading enzymes (CWDEs) to degrade host plant cell walls for invasion. We submitted secretomic and transcriptomic data to the Carbohydrate‐Active EnZymes database (http://www.cazy.org/) to screen the CWDEs secreted by *P. citricarpa*. We identified and divided a set of 81 annotated DEPs/DEGs into five classes on the basis of enzymatic activities: 37 belonged to the glycoside hydrolase (GH) family that hydrolyses the glycosidic bond between two or more carbohydrates; 39 contained carbohydrate‐binding modules for cellulose binding; 25 are members of carbohydrate esterases; 11 belonged to the auxiliary activity family; and the two remaining CWDEs belonged to the glycosyltransferase family (Table S4). Of the CWDEs, those that degrade pectin, cellulose and hemicellulose were often highly expressed. Interestingly, 16 CWDEs were significantly upregulated on the mRNA and secretary protein levels (Table 1 and Fig. 5).

**Table 1:** Crucial pathogenicity factors predicted by carbohydrate‐active EnZymes database and PHI database in *Pseudofabraea citricarpa*.

ID	Protein symbol	CAZy description; CAZy level B	PHI description	Abundance (log_2_ ratio)	
				Transcript	Protein
Pscit00856	chi3	Chitinase; GH18	–	3.03	0.88
Pscit00870	mns1B	Hypothetical protein; GH47	–	1.12	1.04
Pscit01321	plyA	Hypothetical protein; GH134, GH36	Pectate lyase	5.33	1.30
Pscit01610	aspnd1	–	Effector	1.03	0.57
Pscit01740	–	Unnamed protein product; CBM52	Effector	2.34	0.78
Pscit01800	celB	Glycoside hydrolase family 5 protein; CBM1	Effector	1.03	1.78
Pscit02757	asl1	Uncharacterized protein; GH128	–	1.45	0.70
Pscit02908	cbhB	Glycoside hydrolase 7; CBM1, GH7	Endo‐β‐1‐related protein	7.42	0.54
Pscit03074	eglD	Similar to endoglucanase II; AA9, CBM1	–	4.28	2.11
Pscit03122	xlnB	Unnamed protein product, partial; GH11	–	2.20	1.08
Pscit03132	plyE	Putative pectate lyase; CBM1	Pectate lyase	2.32	1.25
Pscit03835	melC2	–	–	2.96	0.40
Pscit04256	CYB2	–	Lactate dehydrogenase	2.26	0.54
Pscit04462	galA	Glycoside hydrolase family 53 protein; GH53	–	1.46	1.50
Pscit05560	bglA	Glycoside hydrolase family 3 protein; GH3	Avenacinase	1.59	0.71
Pscit05764	–	Glycosyl hydrolase; CBM13	–	1.37	0.87
Pscit07972	plyA	–	Pectate lyase	1.87	0.69
Pscit10542	cel61a	Glycoside hydrolase family 61 protein; AA9	Transcription factor	2.28	0.87
Pscit10598	–	Unnamed protein product; CBM35, GH43		1.62	0.46
Pscit14040	–	–	Appressorial‐penetration‐related protein	1.16	0.60
Pscit15364	gh5‐1	Probable cellulase precursor; CBM1	Effector	5.82	1.67

#### Virulence factors of *P. citricarpa* screened from PHI‐base

PHI‐base is a manually curated multispecies database combining peer‐reviewed published phenotype data from plant and animal pathogens and gene/protein information (Urban *et al*., 2015a,2015b). We used PHI‐base (www.phi-base.org) to further identify the virulence factors of *P. citricarpa*. Among the 99 DEPs/DEGs with consistent expression, only 13 genes/proteins were homologous to virulence factors from various fungi (Fig. 5). These candidate virulence factors were annotated as effectors (Pscit01610, Pscit01740, Pscit01800 and Pscit15364), pectate lyases (Pscit01321, Pscit03132 and Pscit07972), transcription factor (Pscit10542), avenacinase (Pscit05560), lactate dehydrogenase (Pscit04256), appressorial‐penetration‐related protein (Pscit14040), a endo‐β‐1‐related protein (Pscit02908) and unknown functional protein (Pscit03835) (Tables 1 and S4). Remarkably, the annotation of eight screened DEPs/DEGs from PHI‐base and CAZy database (Fig. 5) indicated that these CWDEs are the putative key virulence factors of *P. citricarpa*.

#### Small cysteine‐rich secreted proteins (SCRSPs)

SCRSPs are secreted directly into host plant cells and are necessary for pathogenicity (Zeng *et al*., 2018). CCD analysis predicted 17 potential SCRSPs with sizes of 94 aa to 194 aa from transcriptomic data (Table 2). All of these SCRSPs were annotated in GenBank. Interestingly, two proteins (Pscit09319 and Pscit09407) were catalogued into the common in fungal extracellular membrane (CFEM) domain, one protein (Pscit02694) was catalogued into the lysin motif (LysM) domain protein, and three proteins were catalogued as host CWDE‐related proteins (Pscit05099, Pscit06657 and Pscit06942). Among these candidate SCRSPs, however, only Pscit03554 (described as the microbial RNases superfamily) was upregulated on the mRNA and protein levels; additionally, four genes (Pscit02422, Pscit06657, Pscit06942 and Pscit12385) were considerably upregulated on the mRNA level.

**Table 2:** Results of the conserved domain search for predicted small cysteine‐rich secreted proteins (SCRSPs) in *Pseudofabraea citricarpa* against the CDD database.

ID	Hit type	No. of Cys	Length	Math site	log_2_ ratio	E‐Value	Accession	Description
Pscit00390	Specific	4	132	32–118	−0.99	7.49E–12	cd00920	Cupredoxin
Pscit01571	Superfamily	4	139	21–138	−0.19	1.98E–57	cl06331	Cerato‐platanin superfamily
Pscit01949	Specific	12	94	42–64	−1.23	1.68E–03	pfam00187	Chitin recognition protein_N
	Specific			71–94		4.31E–03	pfam00187	Chitin recognition protein_C
Pscit02422	Specific	4	185	44–143	1.44	2.11E–61	pfam09056	Prokaryotic phospholipase A2
Pscit02694	Specific	4	156	110–152	−1.25	1.69E–06	cd00118	LysM
	Superfamily			39–81		1.20E–05	cl21525	LysM superfamily
Pscit03381	Specific	6	174	61–152	−0.89	1.34E–17	cd01285	Nucleoside deaminase
Pscit03478	Specific	5	127	90–116	0.17	2.19E–04	pfam03966	Trm112p‐like protein
Pscit03554	Superfamily	4	134	31–131	3.61	9.30E–31	cl00212	Microbial RNases superfamily
Pscit04823	Superfamily	13	153	24–64	−0.03	3.13E–03	cl02475	LIM superfamily_C
Pscit05099	Superfamily	6	188	15–185	−0.22	6.05E–04	cl27306	Lytic polysaccharide mono‐oxygenase
Pscit06657	Superfamily	9	116	22–116	1.86	4.50E–26	cl03405	Glycosyl hydrolase family 45
Pscit06942	Specific	4	115	22–55	3.98	2.49E–08	smart00236	Fungus‐type cellulose‐binding domain
Pscit08296	Specific	6	165	25–165	0.85	4.87E–80	cd03470	Rieske_cytochrome_bc1
Pscit09319	Specific	8	194	20–85	1.02	1.87E–13	pfam05730	CFEM domain protein
Pscit09407	Specific	8	119	19–86	0.17	4.94E–05	pfam05730	CFEM domain protein
Pscit10570	Superfamily	5	182	1–182	−1.25	8.31E–67	cl17068	Adenylate forming domain, Class I superfamily
Pscit12385	Specific	5	168	44–166	1.74	3.64E–51	cd00917	Phosphatidylinositol/phosphatidylglycerol transfer protein

### Secondary metabolism genes involved in invasion

**Figure 6:**
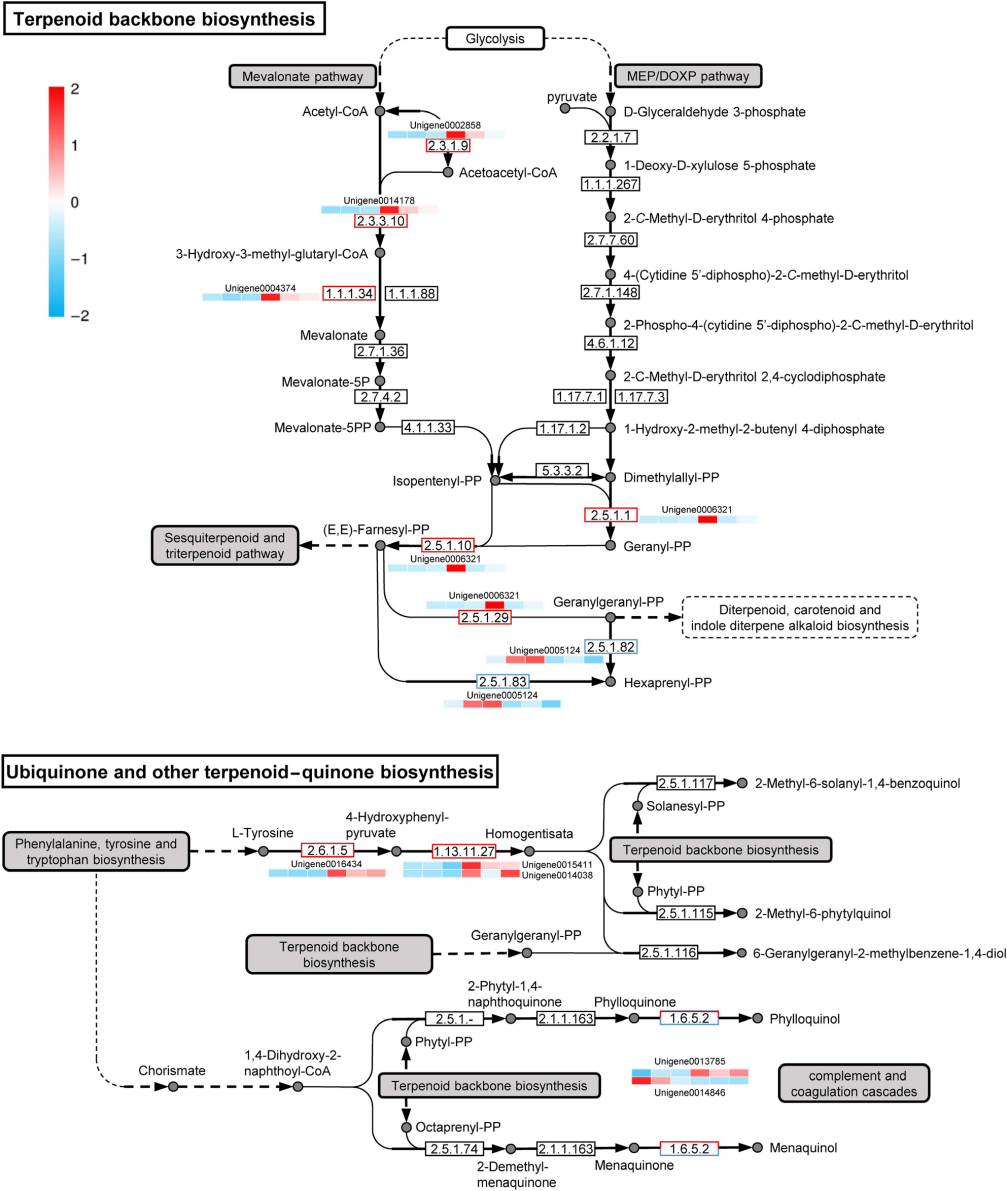
Quantitative transcriptomic analysis of each log_2_ (fold change) values for unigenes encoding enzymes for terpenoid backbone, ubiquinone, and other terpenoid–quinone biosynthetic pathways. Biosynthetic pathways were drawn in accordance with KEGG pathways ko00900 and ko00130 with some modifications. The log_2_(fold change) values in each heatmap are shown from the left in the order of three control treatments and three induced treatments. Red and blue indicate high and low log_2_ (fold change) values respectively. A list of unigenes and their RPKM values are shown in Table S5.

Phytotoxic secondary metabolites, including polyketides, non‐ribosomal peptides, terpenes and alkaloids, are crucial weapons that pathogens employ to kill host cells (Yin *et al*., 2015). We performed KEGG analysis to screen the transcriptomic data for DEGs that encode secondary metabolism core enzymes to identify genes involved in the biosynthesis of secondary metabolites in *P. citricarpa*. We identified 10 core enzyme genes in the *P. citricarpa* transcriptomic. These enzymes were involved in terpenoid backbone biosynthesis (KEGG pathway ko00900) and ubiquinone and other terpenoid–quinone biosynthetic pathways (ko00130) (Table S5). In the terpenoid backbone biosynthesis, only the mevalonate pathway is involved in secondary metabolism associated with infection (Fig. 6). In addition, the expression levels of two polyketide synthases (Pscit02596 and Pscit15312) and one non‐ribosomal peptide synthetase (Pscit07138) were considerably induced. The expression of Pscit07138 was upregulated by more 10‐fold under the induction treatment relative to that under the control treatment (Table S5). However, no alkaloid‐biosynthesis‐related unigene was identified.

## Discussion

The discoveries of mRNA and protein synthetic machinery have provided a powerful foundation for explaining the transfer of information from genes to proteins in molecular terms (Polyansky *et al*., 2013). Integrated secretomic and transcriptomic analyses have enabled the identification of the gene repertoire that is likely involved in host infection and colonization by *P. citricarpa*. PCC is a well‐established measure of correlation and ranges from + 1 (perfect correlation) to −1 (perfect but negative correlation), with 0 denoting the absence of a relationship (Adler and Parmryd, 2010). The PCC value (*r *=* *0.228) obtained in the present study revealed that the overall correlation between secretomic and transcriptomic data was weak, consistent with the familiar study on *Drosophila melanogaster* (Casas‐Vila *et al*., 2017). The weak correlation between secretomic and transcriptomic data may be mainly attributed to the influence and regulation of transcription and translation by various factors that drastically affected the expression level of mRNAs and the accumulation of secreted proteins, underscoring the importance of proteomics to study invasion progression. Nevertheless, several transcripts and their cognate proteins involved in plant cell wall degradation, pathogen–host protein interaction and secondary metabolite biosynthesis were identified as key pathogenicity factors.

Integrated analysis revealed that 81 DEPs/DEGs for CWDEs had the highest expression levels in *P. citricarpa*. Among the DEPs/DEGs involved in the degradation of cellulose, hemicellulose (glycoside hydrolases) and pectin (pectate lyases), 16 exhibited high transcript and cognate secretary protein expression levels under the induction treatment (Table 1 and Fig. 5). The positive correlation between these CWDEs and the virulence of *P. citricarpa* observed in this study corresponds with that observed in studies on the necrotroph *Botrytis cinerea*, the wilt pathogen *Verticillium dahliae* and the blotch fungus *Mycosphaerella graminicola* (Gibson *et al*., 2011). The host cell wall is an important barrier that plants use to defend against attack by phytopathogenic fungi (Underwood, 2012). Pathogenic fungi overcome this barrier by producing CWDEs that destroy the cell wall polymers cellulose, xylan and pectin; CWDEs also play important roles during the late stages of invasion (Zhao *et al*., 2013; Kubicek *et al*., 2014).

Web‐accessible PHI‐base catalogues have been used to experimentally verify pathogenicity, virulence and effector genes from various pathogens (Winnenburg *et al*., 2006; Urban *et al*., 2015a,2015b). We predicted 13 PHI‐related genes/proteins from 99 consistently upregulated DEPs/DEGs (Fig. 5). We annotated four of these DEPs/DEGs as ‘effectors’ by using BLASTp with PHI‐base. ‘Effectors’ are critical components in the secretion of pathogenic proteins required for pathogenesis, plant immunity modulation and infection (Toruno *et al*., 2016). The features of some specific fungal effectors have been reported (Sperschneider *et al*., 2016), and we have successfully identified several pathogen‐related effector proteins from fungal transcriptomic and secretomic data. Additionally, the highly expressed Pscit01800 was annotated as a GH family 5 protein in the CAZy database and as an effector in the PHI‐base (Fig. S7). Swiss‐Prot annotation showed that Pscit01800 is an endoglucanase. β‐1, 4‐endoglucanases (cellulases) are produced by numerous plant pathogens to degrade cellulose, a major component of plant cell walls (Kikuchi *et al*., 2004). Similar to the GH12 glycosyl hydrolase XEG1 of *Phytophthora sojae* (Ma *et al*., 2015) and T6SS GH effector‐immunity families of *Pseudomonas protegens* (Whitney *et al*., 2013), which act as the key virulence determinants of pathogens and effectors that are recognized via the plant's PAMP recognition machinery, β‐1, 4‐endoglucanases play a dual role in pathogenesis.

*Small cysteine‐rich secreted proteins* are secreted directly into host plant cells and perform multiple biological functions, such as host recognition or colonization, and participate in the generation of hypersensitive responses to induction and pathogenicity (Zeng *et al*., 2018). In this study, we predicted 17 potential SCRSPs on the transcript level. Only Pscit03554 was upregulated and provided in transcriptomic and secretomic data. Blast and conserved domain search results revealed that this unigene is a ribonuclease‐domain‐containing protein (Fig. S8) and is a putative enzyme that could be induced by RNA or other compounds. This extracellular RNase likely contributes to the digestion of polyribonucleotides in host cells for the provision of diffusible nutrients for fungal cell growth (Egami and Nakamura, 1969). Additionally, we found that Pscit09319 and Pscit09407 contain CFEM domains, which typically have eight cysteine residues, and are fungal‐specific extracellular membrane proteins similar to Pth11p of *Magnaporthe grisea*. Pth11p plays important roles in appressorium formation and fungal pathogenesis (DeZwaan *et al*., 1999). A LysM containing protein Pscit02694 was also identified in our study. In phytopathogenic fungi, conserved LysM proteins have been characterized as effectors to perturb chitin‐induced immunity in plant hosts (Kombrink and Thomma, 2013; Cen *et al*., 2017), indicating Pscit02694 is likely to contribute to fungal virulence. Therefore, the SCRSPs predicted in *P. citricarpa* may also have key functions in pathogenesis.

Our analytical results for secondary metabolism genes revealed that the pathways of terpenoid backbone biosynthesis (ko00900) and ubiquinone and other terpenoid–quinone biosynthesis (ko00130) are associated with pathogenesis (Fig. 6). The drastically upregulated expression levels of five unigenes in the mevalonate pathway relative to those in the MEP/DOXP pathway revealed that the former pathway is the more important than the latter pathway in terpenoid backbone biosynthesis. Five additional regulated unigenes are located in the ubiquinone and other terpenoid–quinone biosynthesis pathways with the closest relationship with terpenoid backbone biosynthesis. The mevalonate pathway begins with acetyl‐CoA and ends with the production of isopentenyl pyrophosphate and dimethylallyl pyrophosphate, which are used to synthesize terpenoids, a large and diverse class of naturally occurring organic chemicals derived from terpenes (Holstein and Hohl, 2004). The ko00900 and ko00130 pathways could then influence downstream secondary metabolism pathways, such as the sesquiterpenoid and triterpenoid pathway (ko00909); the diterpenoid biosynthesis pathway (ko00904); the carotenoid biosynthesis pathway (ko00906); the indole diterpene alkaloid biosynthesis pathway (ko00403); and the phenylalanine, tyrosine and tryptophan biosynthesis pathway (ko00400) (Fig. 6). Thus, terpenes produced by *P. citricarpa* and not other secondary metabolites may play a core role during invasion. This interpretation corresponds with that of a previous study on *B. cinerea* that demonstrated the considerable phytotoxic activity of terpenes against host leaves and intact plants (Gonzalez Collado *et al*., 2007).

**Figure 7:**
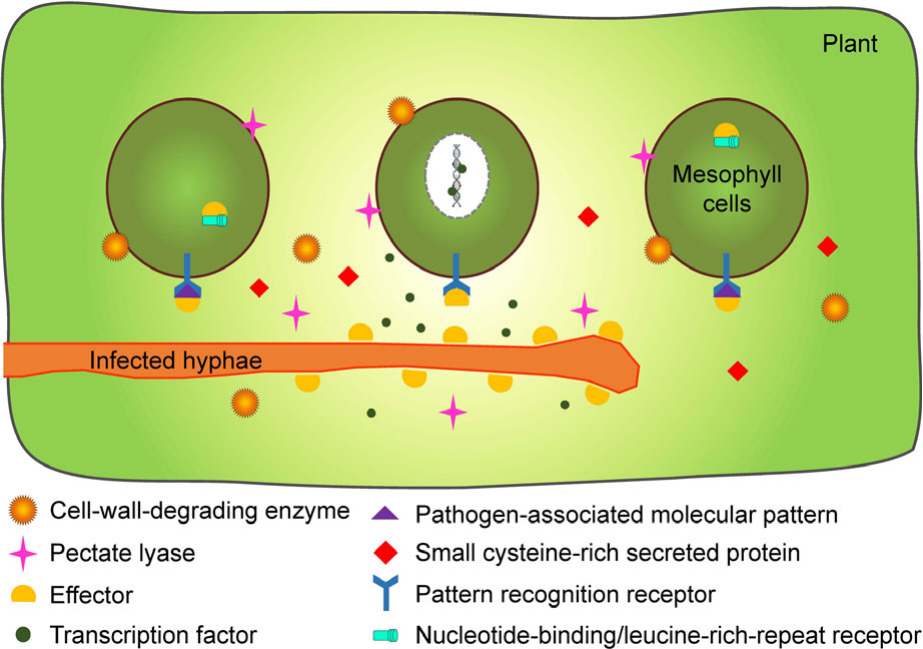
Overview of diverse roles that the critical virulence factors in *Pseudofabraea citricarpa* associated with pathogenicity. Fungal cell‐wall‐degrading enzymes and pectate lyases are involved in plant cell wall degradation. Fungal effectors may combine with fungal pathogen‐associated molecular patterns (PAMPs) so that suppress PAMP‐triggered immunity transduced by plant pattern recognition receptors, or interfere with host intracellular receptors that lead to effector‐triggered susceptibility. Fungal transcription factors could bind to plant defence‐related DNA sequences for perturbing defence signalling transduction. Some fungal small cysteine‐rich secreted proteins may also have key functions in pathogenesis.

We applied an integrated transcriptomic and secretomic analysis approach to identify the pivotal virulence factors of *P. citricarpa*. Using this pipeline, we identified 99 consistently expressed DEPs and DEGs that were mainly involved in glycosidic hydrolysis and energy metabolism, secondary metabolite biosynthesis, glycolysis and aromatic compound degradation. In addition, we identified numerous potential key pathogenicity factors associated with plant cell wall degradation and plant–pathogen protein/polyribonucleotide interaction (Fig. 7). Lastly, we illustrated that most genes belonging to the terpene biosynthesis pathway, which is homologous in other pathogens have demonstrated involvement in pathogenicity, were highly expressed. Our findings will facilitate functional fungal gene and secretory protein studies, improve the current understanding of the participation of secretory proteins in citrus–pathogen interactions in *P. citricarpa*, and consequently help further exploit specific biocontrol agents against *P. citricarpa*, leading to positive environmental, social and economic outcomes (Pandin *et al*., 2017; Trivedi *et al*., 2017).

## Experimental procedures

### Fungal culture conditions and sample collection

The *P. citricarpa* strain used in this study was isolated from Satsuma mandarin leaves with disease symptoms and was maintained on PDA plates at 20°C (Yang *et al*., 2018). For the determination the growth curves of *P. citricarpa*, five mycelium PDA discs with diameters of 0.5 cm were inoculated into Erlenmeyer flasks containing 200 ml of PDB medium. The Erlenmeyer flasks were agitated at 120 rpm at 10°C for 22 days. Mycelia were collected after each 3 days, dried and weighed for growth curve construction. Three biological replicates were performed at each time point.

Fresh Satsuma mandarin leaf tissue was ground to fine flour under liquid nitrogen for inducing the expression of pathogenicity genes of *P. citricarpa* as described previously (Yang *et al*., 2012; Manikandan *et al*., 2018). For RNA extraction, five replicates were cultured at 10°C for 17 days in 200 ml of medium containing 2 g of finely leaf flour. Then, mycelia were harvested through centrifugation at 3750 rpm and 4°C for 20 min and stored at −80°C. All experiments were performed in triplicate. Three controls without leaf powder were used.

Medium supernatants were centrifuged at 10 000 rpm and 4°C for 10 min for secretory protein extraction. Then, residual mycelia and medium were removed by using a 0.45 μm filter. Filtered culture supernatants were extracted by using the modified DOC–TCA protein precipitation procedure for secretory protein purification (Schwarz *et al*., 2007). DOC powder was added to the filtered supernatant at the final concentration of 0.02% (w/v). The mixture was then incubated on ice for 30 min. TCA was added to the mixture at the final concentration of 10% (w/v). The mixture was then stirred overnight at 4°C. The samples were centrifuged for 20 min at 14 000 rpm and 4°C. Protein pellets were washed twice with ice‐cold acetone, air‐dried and vacuum‐dried for 30 min in a freeze vacuum dryer (precooled 30 min in advance). The obtained protein powders were stored at −80°C.

### secretomic analysis of *P. citricarpa*

For protein quantification, *s*ecretory protein samples were transferred to lysis buffer (2% SDS, 7 M of urea and 1 mg ml^−1^ of protease inhibitor cocktail) and homogenized for 3 min on ice by using an ultrasonic homogenizer. The homogenate was centrifuged at 15 000 rpm for 15 min at 4°C. The supernatant was then collected. Three independent biological replicates were performed per experiment to account for biological variation and ensure that only reproducible responses to treatments were selected for analysis.

### Protein digestion and iTRAQ labelling

iTRAQ analysis was performed at Guangzhou Genedenovo Biotechnology Co., Ltd. (Guangzhou, China). The total protein volume (100 μg) per treatment was adjusted to the final volume of 100 μl with 8 M of urea. Next, 11 μl of 1 M DTT was added to each sample. The samples were incubated at 37°C for 1 h. The samples were centrifuged at 14 000 rpm for 10 min in a 10K ultrafiltration tube (Millipore, Bedford, MA, USA). Then, 120 μl of 55 mM iodoacetamide was added to the sample. The sample was incubated for 20 min under protection from light at room temperature. Each sample was centrifuged thrice to replace 8 M of urea with 10 M of TEAB. Proteins were digested with a sequence‐grade modified trypsin (Promega, Madison, WI, USA) at 37°C overnight. After trypsin digestion, peptides were centrifuged at 13 500 rpm for 12 min, dried through vacuum centrifugation and then labelled with iTRAQ/TMT tags (iTRAQ Reagents‐8Plex (SCIEX)) for 2 h at room temperature. The labelled samples were combined, vacuum‐dried, redissolved in buffer A (20 mM ammonium formate in water, pH adjusted to 10.0 with ammonium hydroxide) and fractionated through high‐pH separation using the Ultimate 3000 System (Thermo Fisher Scientific, Milford, MA, USA) connected to a reverse‐phase column (XBridge C18 column, 4.6 mm × 250 mm, 5 μm, Waters Corporation, Milford, MA, USA). High‐pH preseparation was performed using a linear gradient starting from 5% B to 45% B in 40 min. Twelve fractions were collected. Each fraction was dried in a vacuum concentrator for the next step.

### Nano‐HPLC‐MS/MS analysis

Resuspended peptide fractions were separated through nanoLC and analysed by using online electrospray tandem mass spectrometry with an Easy‐nLC 1000 system (Thermo Fisher Scientific) connected to an Orbitrap Fusion Tribrid mass spectrometer (Thermo Fisher Scientific) equipped with an online nanoelectrospray ion source. Furthermore, 10 μl of peptide sample was loaded onto the trap column (Thermo Scientific Acclaim PepMap C18, 100 μm × 2 cm) at the flow rate of 10 μl/min for 3 min and subsequently separated on an analytical column (Acclaim PepMap C18, 75 μm × 15 cm) with a linear gradient. The column flow rate was maintained at 300 nl/min. The electrospray voltage applied was 2 kV. The fusion mass spectrometer was operated in data‐dependent acquisition mode to switch automatically between MS and MS/MS acquisition. Full‐scan MS spectra (m/z 350–1550) were acquired with a mass resolution of 120K. Sequential high‐energy collisional dissociation MS/MS scans with a resolution of 30K were then performed. Intense signals in the MS spectra (> 1e4) were subjected to additional MS/MS analysis. The automatic gain controls for the MS and MS/MS were set as 4e5 and 8e4 respectively. The maximum ion injection times for the MS and MS/MS were 50 and 100 ms respectively. The isolation window was set as 1.6 Da. In all cases, one microscan was recorded using a dynamic exclusion of 30 s. A cut‐off of a fold change > 1.2 (*P *<* *0.05) was used to identify differentially expressed proteins (DEPs) between treatments.

### Transcriptomic analysis of *P. citricarpa*

Total RNA was extracted from mycelia collected from three independent biological replicate cultures. The mycelia were ground in liquid nitrogen and purified with RNeasy Mini Kit (Qiagen, Hilden, Germany). After the extraction of total RNA, eukaryotic mRNA was enriched with Oligo (dT) beads. Then, the cDNA library was synthesized, and the reactions were sequenced. PCR was amplified and sequenced by Guangzhou Genedenovo Biotechnology Co., Ltd. (Guangzhou, China) using Illumina HiSeq 4000.

Thereafter, adaptor sequences, reads with unknown bases and low‐quality reads were removed from the raw sequenced reads. Clean reads were quantified using the Reads Per kb per Million (RPKM) method (Mortazavi *et al*., 2008). Gene expression levels calculated through the RPKM method can be directly used to compare the gene expression patterns of samples. Principal component analysis (PCA) was performed with R package models (www.r-project.org) to reveal relationships among samples. edgeR package (www.r-project.org) was used to identify differentially expressed genes (DEGs) between induction and control treatments. A cut‐off of fold change ≥ 2 and a false discovery rate (FDR) < 0.05 were used to identify DEGs between treatments.

### Prediction of virulence‐related proteins

Phytopathogenic fungal extracellular carbohydrate‐active enzymes (CAZymes) help break down host cell wall components, such as complex carbohydrates, to enable pathogen access and facilitate infection (Zeng *et al*., 2018). DEG data were submitted to the CAZymes database (http://www.cazy.org/) to search for secretory proteins with an e‐value cut‐off of 10^−10^ for the identification of the clustering of CAZyme‐encoding genes involved in pathogenicity (Cantarel *et al*., 2009).

Pathogen–host interaction (PHI) partners were identified by subjecting predicted secretory proteins to BLASTp against the PHI database (http://www.phi-base.org/) with an e‐value cut‐off of 10^−10^ (Urban *et al*., 2017).

SCRSPs were predicted on the basis of their expected sequence characteristics of < 200 aa residues with an N‐terminal signal peptide and at least four cysteine residues (Zeng *et al*., 2018). Secreted *P. citricarpa* proteins with these characteristics were identified as putative SCRSPs. The conserved domains of SCRSPs were searched by using an online tool Conserved Domain Database (CDD) (https://www.ncbi.nlm.nih.gov/Structure/cdd/wrpsb.cgi) with an e‐value cut‐off of 0.01 (Marchler‐Bauer *et al*., 2011).

Phytotoxic secondary metabolites include polyketides, non‐ribosomal peptides, terpenes and alkaloids (Brakhage, 2013). Genes associated with secondary metabolism were identified on the basis of KEGG metabolic pathways involved in the metabolism of terpenoids and polyketides (ko00900, ko00902, ko00909 and ko00904), as well as ubiquinone and other terpenoid–quinone biosynthetic pathways (ko00130).

## Conflict of interests

None declared.

## Author contributions

Y.H.Y. and C.Z. designed the research. Y.H.Y., A.F. and Y.Y. performed the experiments. Y.H.Y., Y.Y. and C.W.B. analysed the data and wrote the manuscript.

## Supporting information


**Fig. S1.** Growth curve of *Pseudofabraea citricarpa* in terms of dry weight for PDB medium.

**Fig. S2.** Sodium dodecyl sulfate‐polyacrylamide gel electrophoresis of extracted secretory proteins of *Pseudofabraea citricarpa*.

**Fig. S3.** Gene Ontology annotation result of the differentially expressed proteins.

**Fig. S4.** Kyoto Encyclopedia of Genes and Genomes pathway analysis of the differentially accumulated proteins.

**Fig. S5.** Gene Ontology annotation result of the differentially expressed genes.

**Fig. S6.** Kyoto Encyclopedia of Genes and Genomes pathway analysis of the differentially accumulated genes.

**Fig. S7.** SmartBLAST result of Pscit01800.

**Fig. S8.** SmartBLAST result of Pscit03554.

**Table S1.** List of differentially abundant proteins identified by nano‐HPLC‐MS/MS in *Pseudofabraea citricarpa*.

**Table S2.** List of differentially abundant genes identified by RNA‐seq in *Pseudofabraea citricarpa*.

**Table S3.** List of integrated differentially transcripts and their cognate proteins in *Pseudofabraea citricarpa*.

**Table S4.** List of the carbohydrate‐active enzymes and pathogen‐host interaction related proteins predicted by Carbohydrate‐Active EnZymes database and PHI database in *Pseudofabraea citricarpa*.

**Table S5.** List of the secondary metabolism related genes predicted by KEGG pathway annotation in *Pseudofabraea citricarpa*.
