# Supplementary figures and images for: Integrated transcriptomic and secretomic approaches reveal critical pathogenicity factors in Pseudofabraea citricarpa inciting citrus target spot
Source: Microb Biotechnol. 2019 Jun 4;12(6):1260–73. doi: 10.1111/1751-7915.13440 (PMC6801157; doi:10.1111/1751-7915.13440)

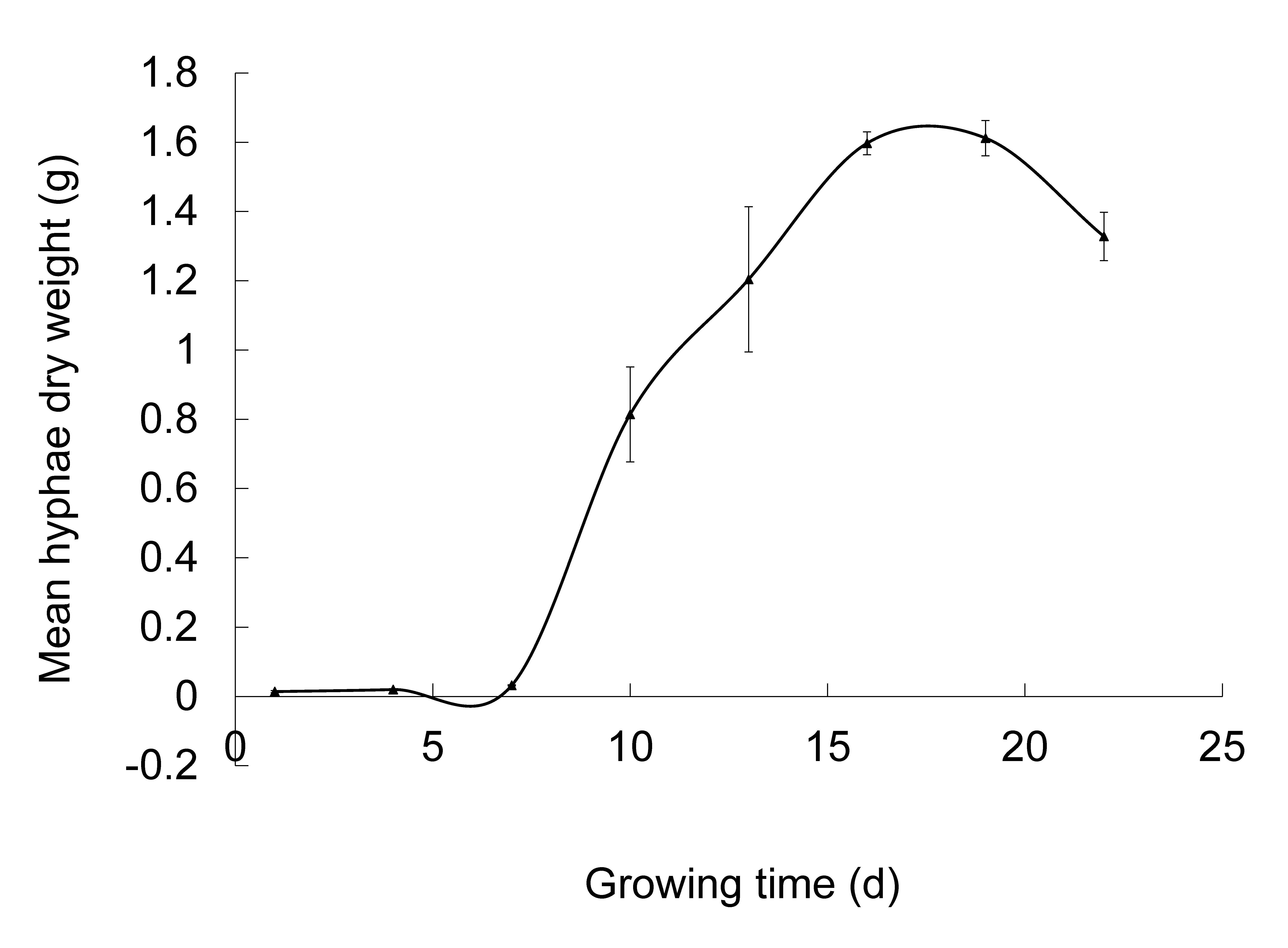

Supplement: Supplementary file 1 — Fig. S1. Growth curve of Pseudofabraea citricarpa in terms of dry weight for PDB medium. [file MBT2-12-1260-s001.jpg]

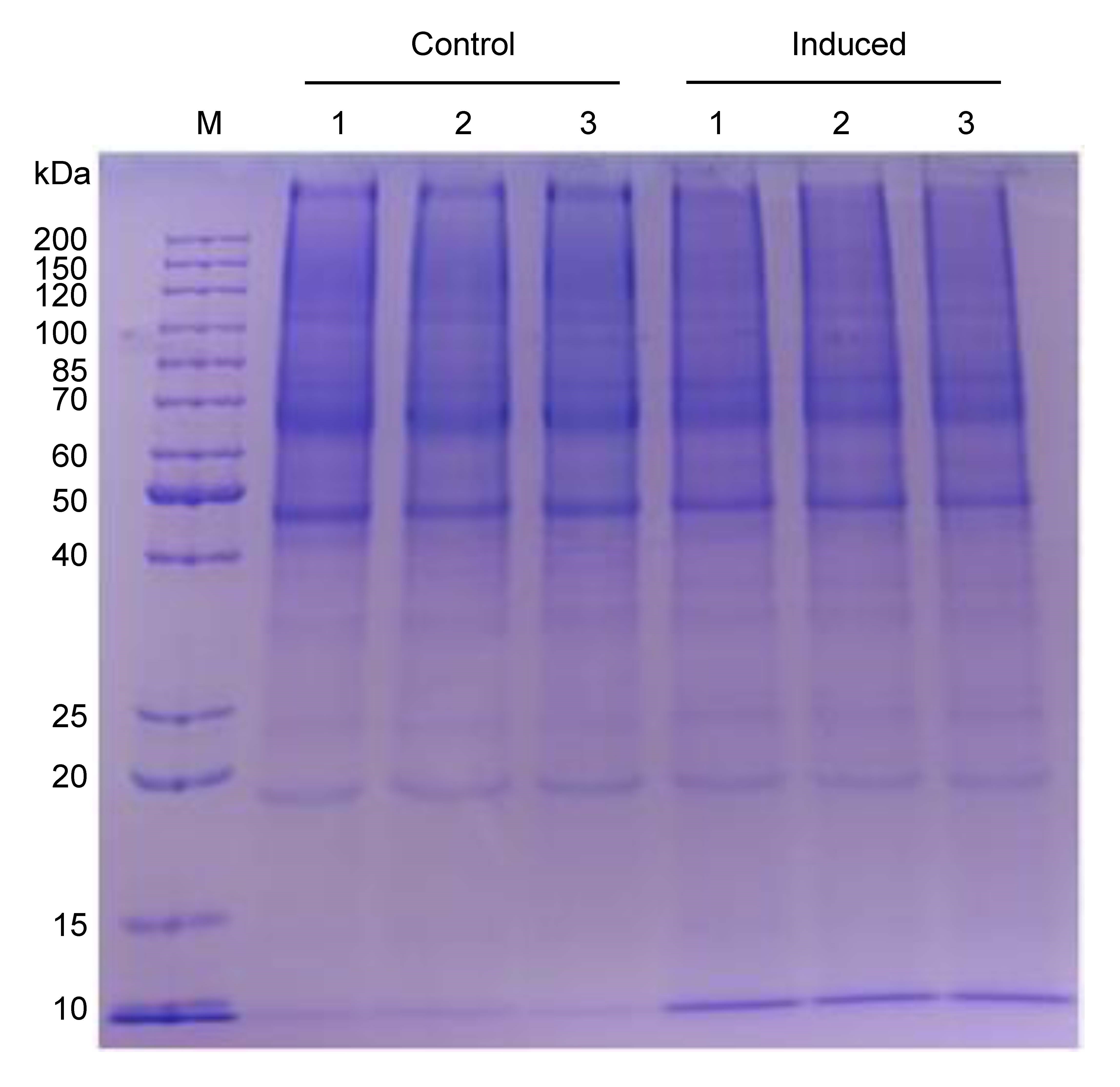

Supplement: Supplementary file 2 — Fig. S2. Sodium dodecyl sulfate‐polyacrylamide gel electrophoresis of extracted secretory proteins of Pseudofabraea citricarpa. [file MBT2-12-1260-s002.jpg]

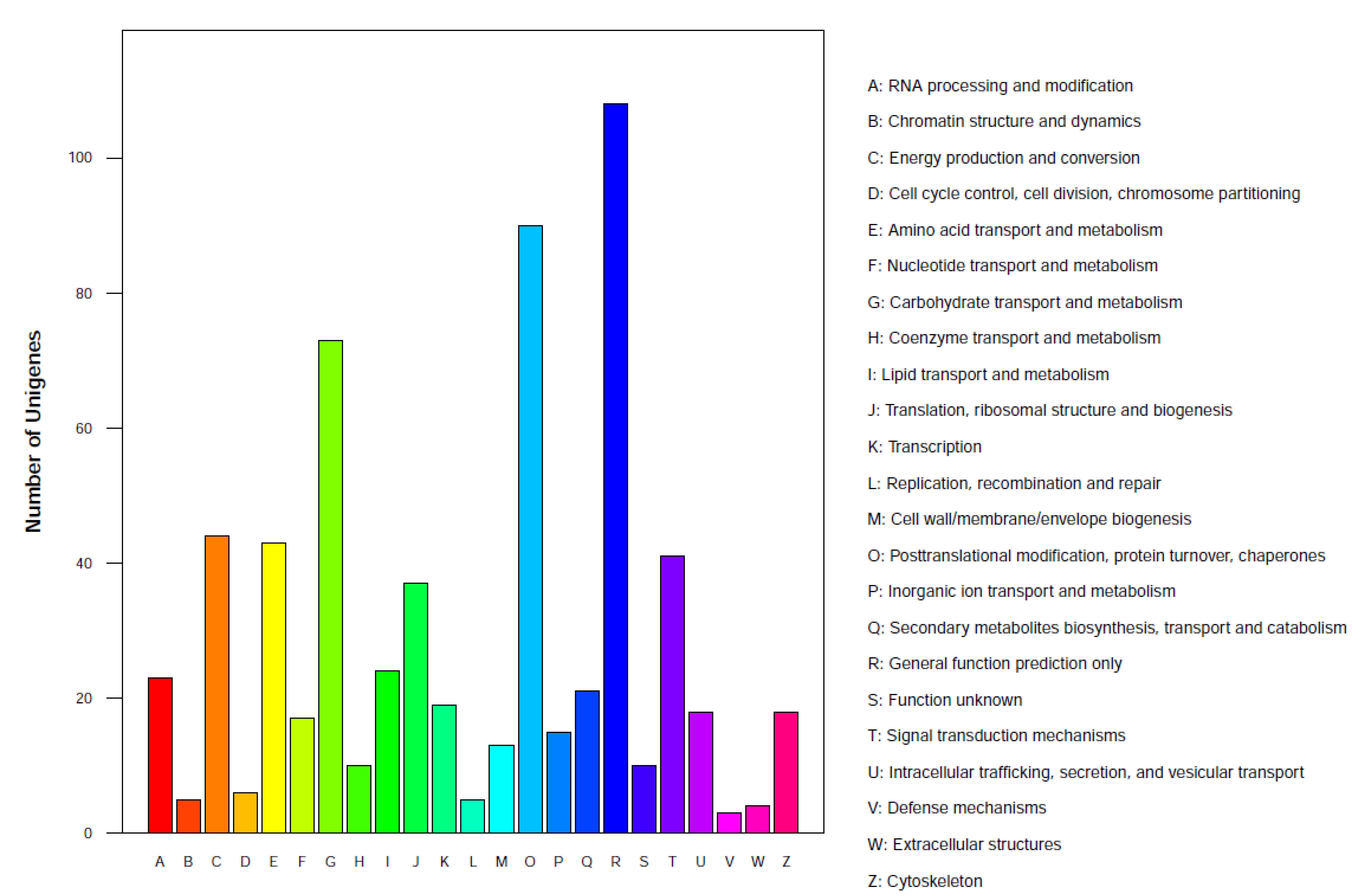

Supplement: Supplementary file 4 — Fig. S4. Kyoto Encyclopedia of Genes and Genomes pathway analysis of the differentially accumulated proteins. [file MBT2-12-1260-s004.jpg]

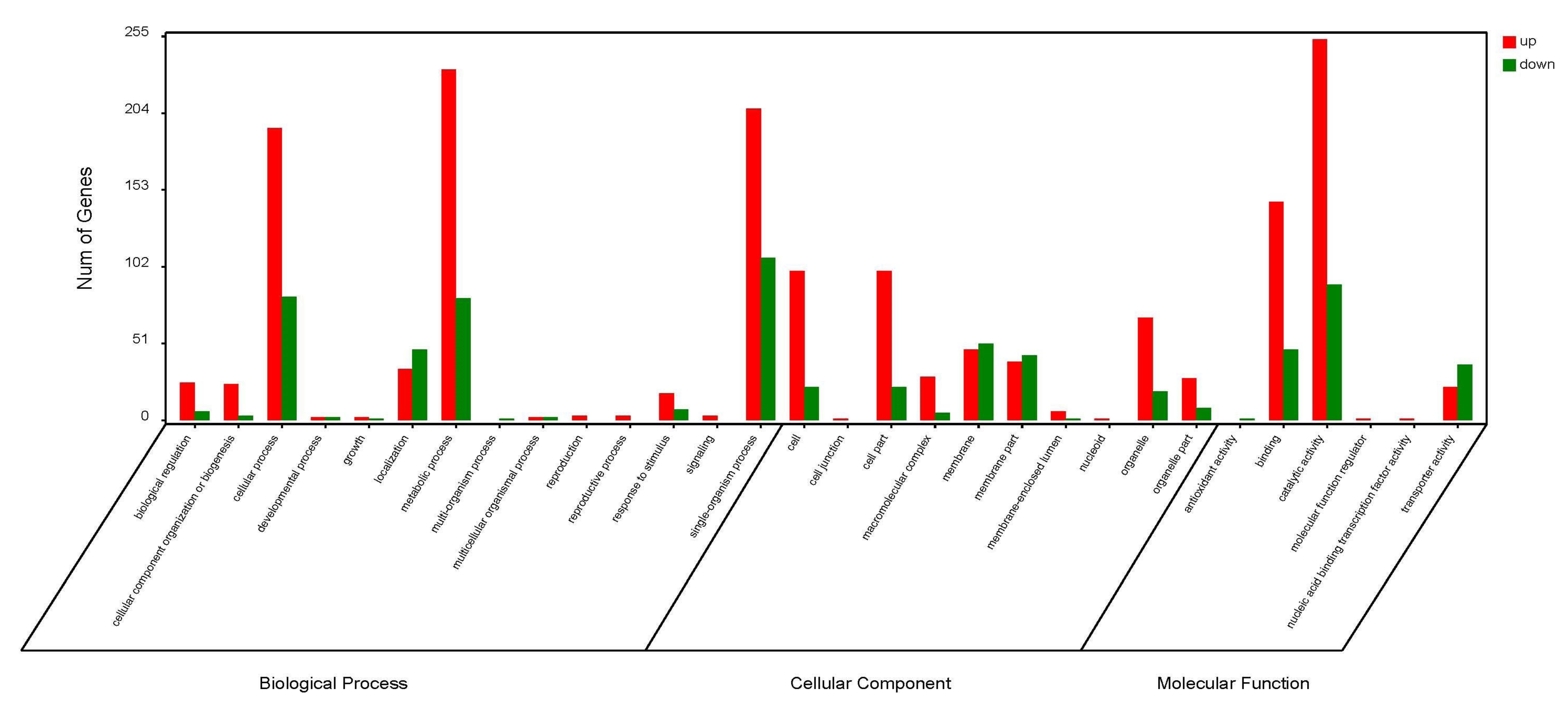

Supplement: Supplementary file 5 — Fig. S5. Gene Ontology annotation result of the differentially expressed genes. [file MBT2-12-1260-s005.jpg]

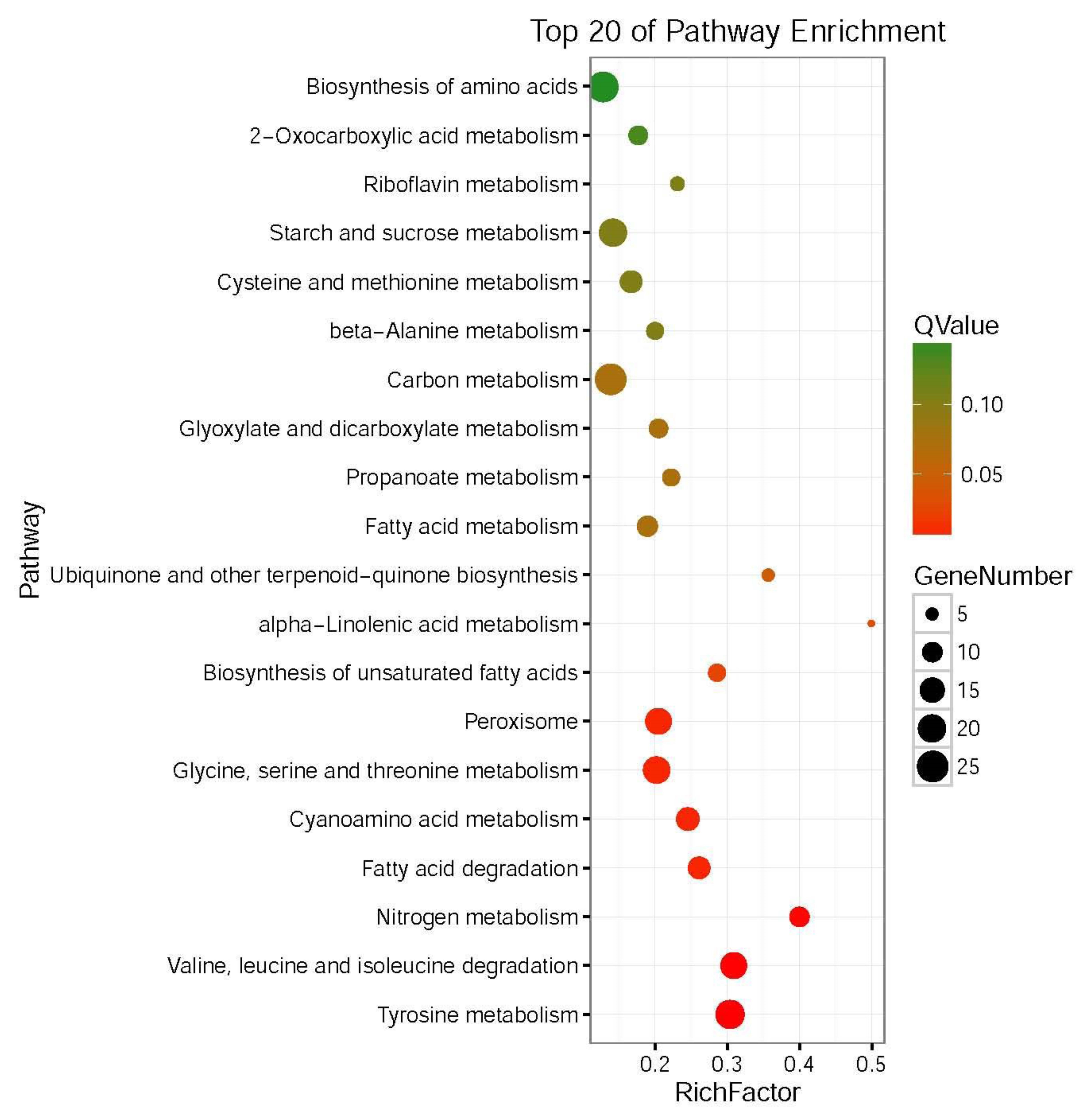

Supplement: Supplementary file 6 — Fig. S6. Kyoto Encyclopedia of Genes and Genomes pathway analysis of the differentially accumulated genes. [file MBT2-12-1260-s006.jpg]

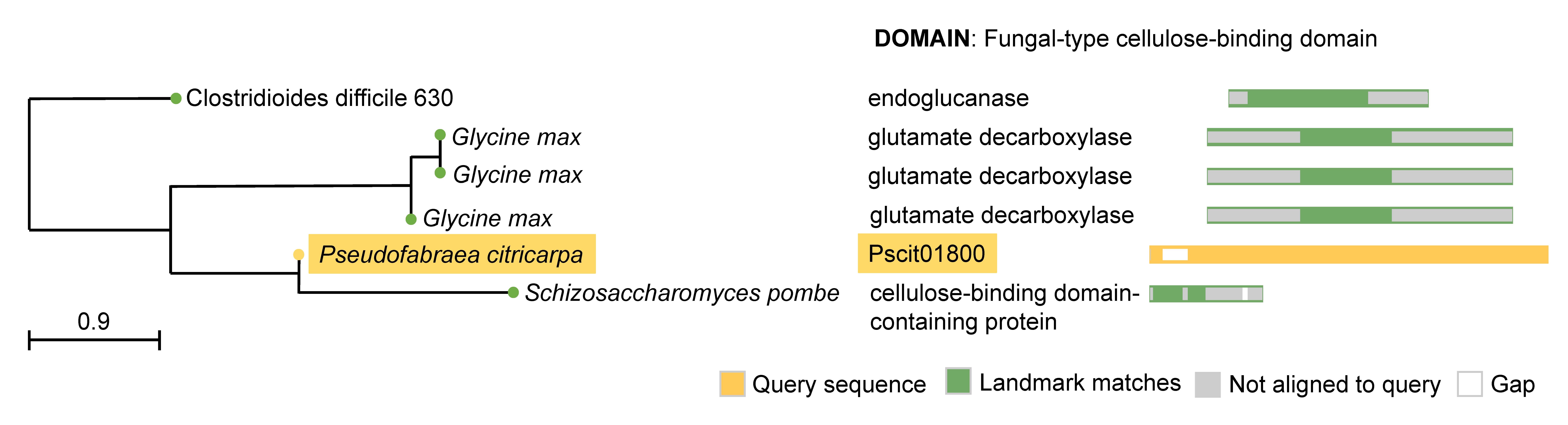

Supplement: Supplementary file 7 — Fig. S7. SmartBLAST result of Pscit01800. [file MBT2-12-1260-s007.jpg]

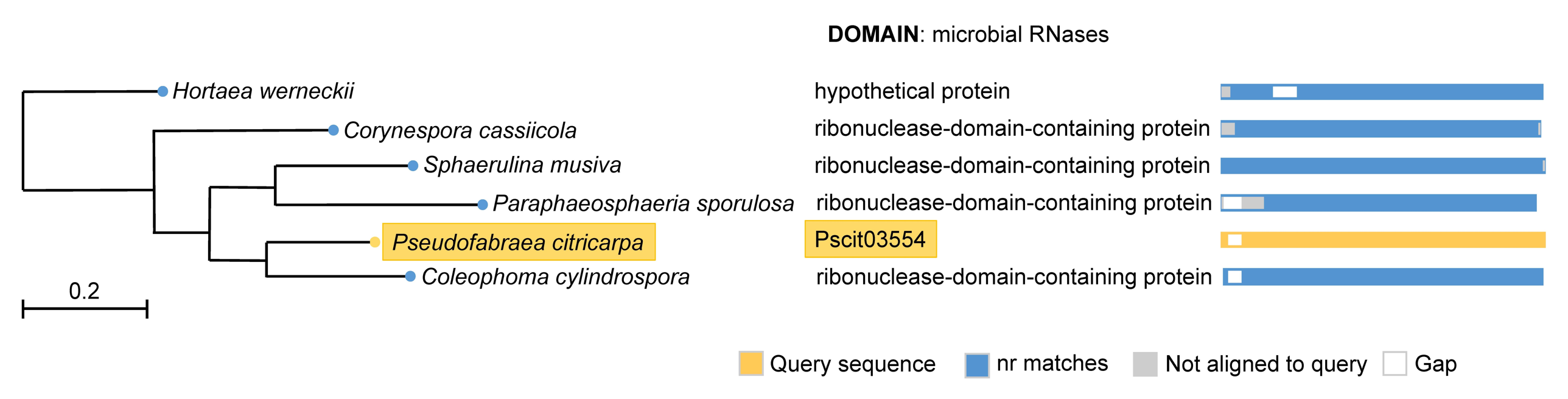

Supplement: Supplementary file 8 — Fig. S8. SmartBLAST result of Pscit03554. [file MBT2-12-1260-s008.jpg]
